# Green Analytical Methods for the Separation of Seven Antihistamines: Application in Separation of Azelastine and Related Impurities in Nasal Solution

**DOI:** 10.1155/2019/9489723

**Published:** 2019-02-11

**Authors:** Lucas Maciel da Costa, Heitor Oliveira de Almeida Leite, Nájla Mohamad Kassab, Anil Kumar Singh

**Affiliations:** ^1^Department of Pharmacy, Faculty of Pharmaceutical Sciences, University of São Paulo, Av. Prof. Lineu Prestes, 580, Cidade Universitária, São Paulo, SP, Brazil; ^2^Graduate Program in Pharmacy, Center for Biological Sciences and Health, Federal University of Mato Grosso do Sul, Brazil

## Abstract

Antihistamines are widely used to alleviate the symptoms caused by allergic reactions. Most of these drugs have zwitteriónicas and/or amphoteric characteristics, which confer additional analytical challenges. This work aimed to develop a single eco-friendly and efficient chromatographic methods for analysis of seven antihistamines, namely, azelastine HCl, desloratadine, ebastine, fexofenadine HCl, ketotifen, loratadine, and olopatadine HCl. The separations were obtained using RP C-18 LUNA (150x4.6mm, 5 *μ*m) column. The mobile phase consisted of acetonitrile and acidified water (pH 2.1) in the following proportion: 15:85, v/v for desloratadine, 25:75, v/v for ketotifen and olopatadine, 32:68, v/v for fexofenadine, 35:65, v/v for azelastine and loratadine, and 45:55, v/v for ebastine. All separations were obtained in less than 7.0 min. A prototype method was fully validated and applied in the assay of azelastine HCl in nasal solutions. The proposed methods for analysis of seven antihistamines are highly efficient, selective, and sensitive. Moreover, all methods can be considered excellent in terms of greenness, with total organic residue < 2.5 mL/analysis. An improved gradient method is also described for separation of azelastine HCl and its related impurities.

## 1. Introduction

It is estimated that around 400 million people are affected by allergic rhinitis and 300 million by asthma in the world. In Brazil, 30 to 35% of the population presents some type of allergic reaction such as allergic rhinitis, asthma, and atopic dermatitis. Total medical expenses exceed 98 million per year with hospitalizations and are the third cause of hospitalization. These overheads outweigh tuberculosis and AIDS [1–3].

The second-generation antihistamines are considered as first-line treatment in allergic disorders and do not cause drowsiness and anticholinergic effects. The improved effectiveness, reduced adverse effects, and remarkable drug compliance of modern antihistaminic drugs may be attributed to low lipophilicity and reduced affinity for brain H1 receptors [4–6].

The second-generation antihistaminic drugs selected in this study are piperidines except for azelastine (methylazepane derivative) and olopatadine (dibenz[b,e]oxepin derivative). The chemical name, molecular structures, and dissociation constants of azelastine HCl (AZE), desloratadine (DESl), ebastine (EB), fexofenadine HCl (FEX), ketotifen (KET), loratadine (LOR), and olopatadine HCl (OLO) are described in Table 1.

Several HPLC methods were found in the scientific literature for quantitative determination of second-generation antihistamines, as a single drug (not cited). The British Pharmacopoeia describe monograph on AZE [7], DESL [8], EB [9], FEX [10], KET [11], and LOR [11] while the United States Pharmacopeia contain monograph on AZE [12], DESL [13], LOR [14], and OLO [15]. The cited monographs also describe separation methods for respective organic impurities. Most of these methods are time-consuming, often present peak tailing, make use of buffers salts, and generate a considerable amount of organic waste.

The United States Pharmacopeia describes chromatographic method for separation of azelastine and its related compounds B, D, and E [12]. The separations were obtained on a cyano HPLC column with mobile phase composed of acetonitrile, monobasic potassium phosphate, and octanesulfonic acid sodium salt. The flow rate was 2.0 mL min^−1^ and detection was made at 210 nm. The system suitability resolution between adjacent peaks is ≥ 1.5 except in case of impurities B and D (≥4.0).

The British Pharmacopoeia [7] and European Pharmacopeia [16] also describe a near identical method for separation of azelastine and its related compounds A, B, C, D, and E.

In the above cases, separations were obtained on a nonconventional column with nitrile groups chemically bonded to porous silica particle with mobile phase composed of buffering and ion pairing salts. Moreover, flow rate is too high, with estimated total run time over 15 minutes.

To our knowledge, no eco-friendly HPLC method was found for separation of seven second-generation antihistamines, with minor modification in mobile phase organic content.

The present study aims to develop a single chromatographic setup for analysis of seven antihistaminic drugs in pharmaceutical formulations. We intended to propose highly efficient and accurate HPLC methods that abide by the green analytical chemistry (GAC) principles. Additionally, a stability-indicating method was developed for separation of azelastine and three related impurities.

## 2. Experimental

### 2.1. Reagents, Sample, and Standard

The certified pharmaceutical secondary standard (assigned purity ≥98%) of azelastine HCl, desloratadine, ebastine, fexofenadine HCl, ketotifen, loratadine, and olopatadine HCl were obtained from Sigma-Aldrich (St. Louis, MA, USA).

The azelastine impurity B (1-benzoyl-2-[(4*RS*)-1-methylhexahydro-1*H*-azepin-4-yl]diazane), azelastine impurity D (4-(4-Chlorobenzyl) phthalazin-1(2*H*)-one), and azelastine impurity E (3-(4-chlorobenzilidene) isobenzofuran-1(3*H*)-one) were obtained from European Pharmacopoeia (EP) Reference Standard Sigma-Aldrich (St. Louis, MA, USA).

The HPLC grade acetonitrile and analytical grade hydrochloric acid (37%) were obtained from Merck KGaA (Darmstadt, Germany). The purified water was obtained from Millipore Milli-Q® Plus (Millipore, Bradford, USA).

The nasal solution containing 0.9 mg mL^−1^ of azelastine was obtained from a local pharmacy. The Rino-Lastin® (Aché Pharmaceutical Laboratories, Brazil) formulation is stated to contain following excipients: benzalkonium chloride, citric acid, sorbitol, hypromellose, purified water, disodium edetate dihydrate, and sodium phosphate dibasic dodecahydrate.

### 2.2. Instrumentation and Chromatographic Conditions

A HPLC system consisted of two solvent delivery pumps, an autoinjector fitted with 20 *μ*L loop, an online degasification system, a column thermostat oven, and an UV/VIS photodiode array detector. The output signal was monitored and integrated using CLASS VP® software (Shimadzu Corporation, Japan).

Analytical conditions were optimized using a Luna-RP C18 column (250x4.6 mm, 5 *μ*m). The mobile phase was constituted of acetonitrile and acidified water (pH 2.1) in the following proportions: 15:85, v/v for DESL, 25:75, v/v for KET and OLO, 32:68, v/v for FEX, 35:65, v/v for AZE and LOR, and 45:55, v/v for EB. The mobile phase flow rate was 1.1 mL/min, and UV detections were made at 220 nm (FEX), 254 nm (EB), 271 nm (LOR), 279 nm (DESL), 287 nm (AZE), and 298 nm (KET and OLO). The volume of injection was fixed at 20 *μ*L.

All analyses were done at room temperature, and the column temperature was controlled at 25°C ± 1. The mobile phase was prepared fresh each day, vacuum-filtered through a 0.45 *μ*m Millipore® (HV) hydrophilic membrane.

### 2.3. Preparation of Standard Solutions

The stock solutions of azelastine HCl reference standard was prepared by transferring mass equivalent to 25 mg of azelastine into a 25 mL volumetric flask. The volume was completed with mobile phase to obtain 1000 *μ*g mL^1^ of azelastine standard stock solution. In case of impurities B, D, and E, 5 mg mass was solubilized in 5 mL of the mobile phase, in separate flasks. All subsequent dilutions were made with mobile phase to obtain target concentrations for analysis and method validation.

The stock solutions of DESL, EB, FEX, KET, LOR, and OLO reference standards were prepared likewise, to obtain 1000 *μ*g mL^1^ of each reference standard, in separate flasks.

### 2.4. Preparation of Sample Stock Solutions

Five AZE nasal solution preparations (claimed 0.9 mg mL^−1^ of AZE) were pooled into a single flask. An aliquot equivalent to 10 mg of AZE was transferred to a 50 mL volumetric flask. The volume was completed with mobile phase to obtain 200.0 *μ*g mL^−1^ of AZE. An ultrasonic water bath was used for 5 min, to aid extraction and solubilization. The resultant sample stock solutions were diluted accordingly and were used to determine the precision and accuracy of the method.

### 2.5. System Suitability

According to official guidelines, the system suitability tests are an integral part of method development [20–22]. Once optimum chromatographic conditions were obtained, a systematic chromatographic analysis of seven antihistaminic drugs was performed. We evaluated capacity factor, retention factors, repeatability, peak asymmetry, and number of theoretical plates. During the process, most appropriate chromatographic conditions were achieved, with acceptable relative standard deviations (RSD), for seven antihistamines, individually.

### 2.6. Validation of the Prototype HPLC-UV Method for Azelastine HCl

To demonstrate adequateness of the proposed methods for seven antihistaminic drugs, azelastine HCl was selected as prototype drug. The proposed method was fully validated according to international guidelines on validation of a chromatographic method for pharmaceutical drug products and applied in the analysis of AZE in nasal solutions [21–24].

The selectivity of the proposed method was evaluated through the analysis of reference standards, sample, and placebo solutions. Besides, peak purity index of obtained chromatograms was accessed by UV diode array detector.

*Linearity*. The mean peak areas (n=3) were plotted against respective concentrations in a range from 10.0 to 60.0 *μ*g mL^−1^ of AZE. The linear regression of the curve was accessed by the least-squares method, at five distinct concentration levels. All solutions were prepared fresh, and dilutions were made with mobile phase (pH=2.1).

(1) $$\begin{array}{c} Y=ax+b \end{array}$$

where *Y* is the peak area; *x* is concentration; *a* is the slope of the curve, and *b* is intercept of the curve on y-axes.

*Precision*. The intra- and interday precision (repeatability) were evaluated by repeated analysis of sample solutions (n=10). Fresh sample solutions containing approximately 40.0 *μ*g mL^−1^ of AZE were analyzed, separately, on three consecutive days. The intra- and interday precisions are expressed as relative standard deviation (RSD) amongst analytical responses [21, 22].

*Accuracy*. The accuracy was determined by recovery of the standard from the sample matrix. The AZE sample solutions were spiked with known amounts of standard solutions, at three concentration levels, in separate flasks. The resultant fortified sample solutions were analyzed by the proposed method. The recovery amount (R) was calculated by comparing the theoretical and found amounts of AZE standards [23].

*Detection Limit (DL) and Quantitation Limits (QL)*. DL and QL were estimated based on the mean standard deviation and the slope of the AZE calibration curve. The estimated values for DL (3 times signal/noise ratio) and QL (10 times signal/noise ratio) were cross-checked by actual analysis of standard solutions.

*Robustness*. The robustness of the proposed method was evaluated by the deliberate modification of chromatographic conditions such as organic content, mobile phase pH, and flow rate. The impact of these changes on the AZE peak area, retention time (t_R_), and tailing factor (T_f_) was analyzed. The chromatographic conditions were modified, individually and separately. The AZE sample solutions were injected into the system (n=3) under univariate conditions and the results were statistically analyzed.

## 3. Results and Discussion

The discovery of third-generation antihistamine drugs has brought additional analytical challenges, besides the therapeutic benefits. However, there is limited information on the related impurities and their probable human cardiotoxic effects and pharmacokinetic and prolonged pharmacodynamics profiles [25, 26].

The third-generation H1 antihistamines have an atypical characteristic in their molecular structures. Depending on the pH of the medium, they often are electrically neutral due to opposite charge at different nonadjacent atoms and/or amphoteric due to the presence of acid and basic groups in their molecular structures [17, 27]. Due to the zwitterionic and/or amphoteric characteristics of these drugs, they often present additional analytical challenges, such as excessive retention and peak tailing due to secondary interactions with the stationary phase [28–31]. The low solubility of these drugs, in reverse phase mode, often impacts adversely on chromatographic separations.

Besides these important aspects, it is necessary to constantly seek fast and alternative methods that increase productivity, reduced solvent, and sample consumption. Most of the solvents used in liquid chromatography are hazardous to analysts and environment due to inherent volatility, flammability, and toxicity of these solvents [18]. The high sensitivity is desirable, and GAC methods are welcome with trace quantities of organic waste [18, 27, 31–33].

### 3.1. Method Development and Optimization

We tested several reverse-phase columns, such as conventional C18 and core-shell technology C18 stationary phase, besides, -C8, -CN, and –NH2 columns. The preliminary chromatographic parameters obtained with the RP C-18 LUNA (250x4.6mm, 5 *μ*m) column were satisfactory. This column was selected for optimization of chromatographic conditions for separation of seven antihistaminic drugs and system suitability tests.

The separations obtained with acetonitrile, as organic phase modifier, were better than those obtained with methanol. Neat improvement in peak symmetry and intensity with acetonitrile can be attributed to the solubility of antihistaminic drugs in acetonitrile [17].

According to the pH dependent solubility curves described in the literature [17] and in-lab experimental work, the solubility of all seven antihistaminic drugs shows remarkable improvement in acidic media (ionic form). For example, AZE is moderately soluble in water at neutral pH (0.5 mg mL^−1^); however at pH 1.7 the value may reach 381.9 mg mL^−1^ [17]. Likewise, a significant improvement in the solubility of DESL, EB, FEX, KET, LOR, and OLO was found at pH ≤ 2.0.

To improve column efficiency and avoid peak tailing during chromatographic runs, in reverse phase mode, it is essential to assure a single ionic form of molecules [29, 31]. The dissociation constants of AZE, DESL, EB, FEX, KET, LOR, and OLO are described in Table 1 [17]. A mobile phase with pH 2.1 was selected to assure near-total ionized forms (more polar microspecies) of cited antihistamines, throughout chromatographic runs. Further, the selected pH may facilitate drug solubilization and mass transfer of selected antihistaminic drugs during chromatographic interactions, in reverse phase mode.

Finally, most of the commercial reverse phase columns are stable at pH between 2.0 and 8.0. Thus, a mobile phase composed of acetonitrile and acidified water (pH 2.1) was selected for further method development and optimization of chromatographic conditions.

### 3.2. System Suitability Test

The seven antihistaminic drugs selected for study are structurally related and belong to the same therapeutic class; however chromatographic interaction with the column is dissimilar. To develop a unique eco-friendly method, factors such as mobile phase organic content, flow rate, and volume of injection were optimized through univariate experimental design approach for seven antihistaminic drugs.

**Figure 1:**
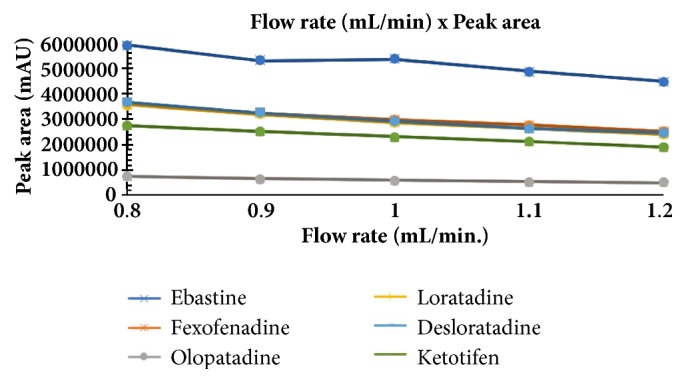
Impact of mobile phase flow rate on peak area of AZE, DESL, EB, FEX, KET, LOR, and OLO.

**Figure 2:**
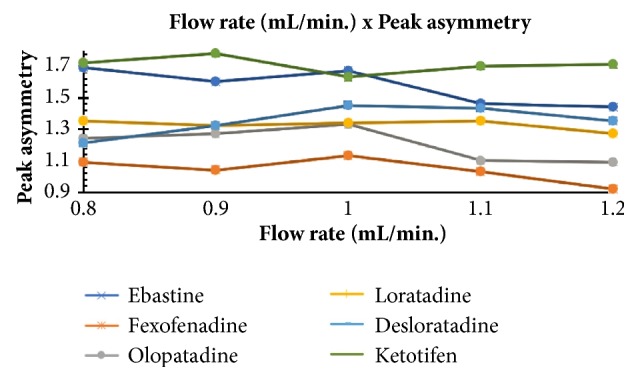
Impact of mobile phase flow rate on peak asymmetry of AZE, DESL, EB, FEX, KET, LOR, and OLO.

**Figure 3:**
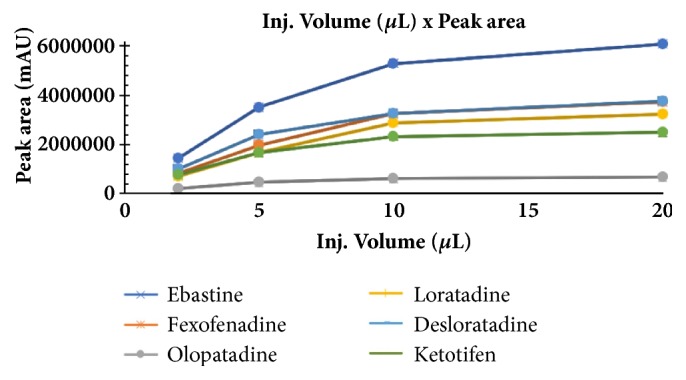
Impact of sample injection volume on peak area of AZE, DESL, EB, FEX, KET, LOR, and OLO.

**Figure 4:**
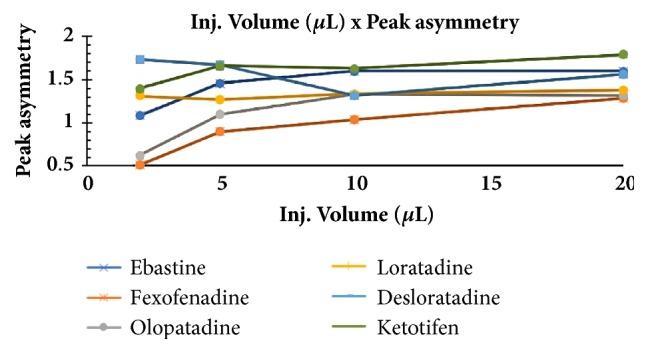
Impact of sample injection volume on peak asymmetry of AZE, DESL, EB, FEX, KET, LOR, and OLO.

The optimum conditions for AZE, DESL, EB, FEX, KET, LOR, and OLO were fixed based on method sensitivity (peak area), efficiency (theoretical plates), asymmetry factor (As), and rapid elution (retention factor). Briefly, standard reference solutions were prepared (200 *μ*g mL^−1^) separately and injected into the system. The organic content of the mobile phase was optimized for rapid elution of antihistamines, separately. In the following step, mobile phase flow rate was adjusted (0.8, 0.9, 1.0, 1.1, and 1.2 mL.min^−1^). The impact of injection volume and mobile phase flow rate on peak areas and asymmetry is shown in Figures 1–4. Finally, injection volume was optimized (2, 5, 10, and 20 *μ*L). Fully optimized conditions for AZE, DESL, EB, FEX, KET, LOR, and OLO are presented in Table 2.

The detection wavelength for AZE, DESL, EB, FEX, KET, LOR, and OLO was selected based on the DAD detector, and the column temperature was maintained at 25°C ± 1.

**Figure 5:**
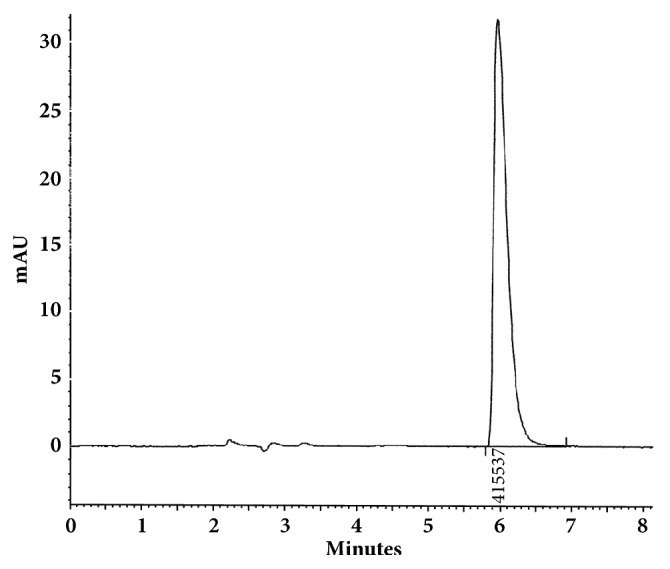
Chromatographic separation of azelastine HCl. Chromatographic conditions described in Table 2.

**Figure 6:**
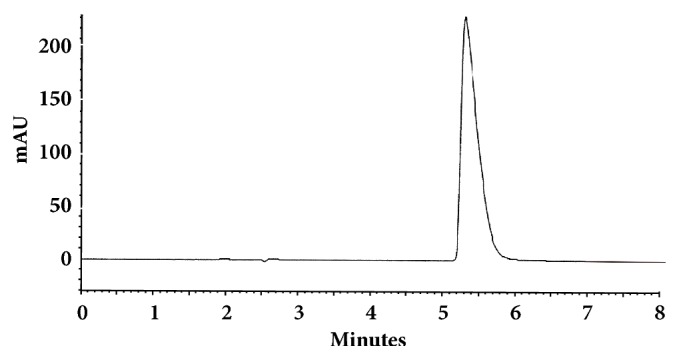
Chromatographic separation of desloratadine. Chromatographic conditions described in Table 2.

**Figure 7:**
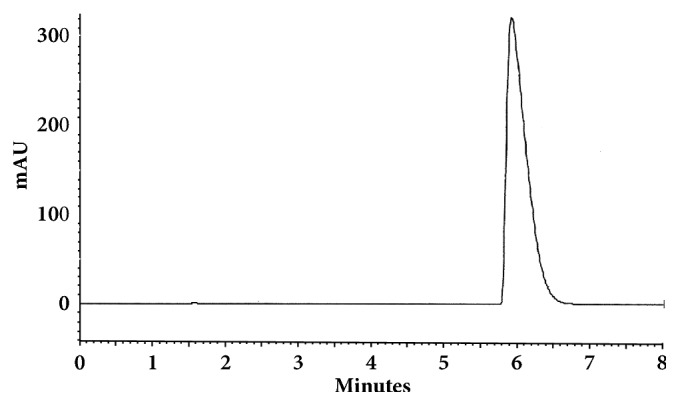
Chromatographic separation of ebastine. Chromatographic conditions described in Table 2.

**Figure 8:**
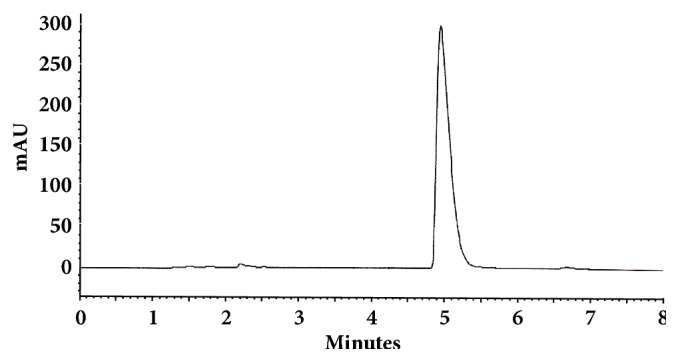
Chromatographic separation of fexofenadine HCl. Chromatographic conditions described in Table 2.

**Figure 9:**
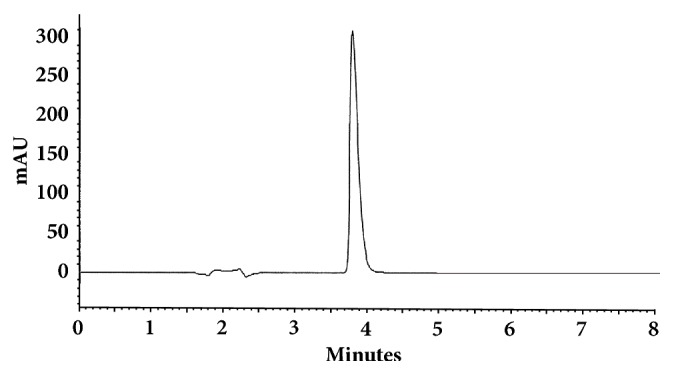
Chromatographic separation of ketotifen. Chromatographic conditions described in Table 2.

**Figure 10:**
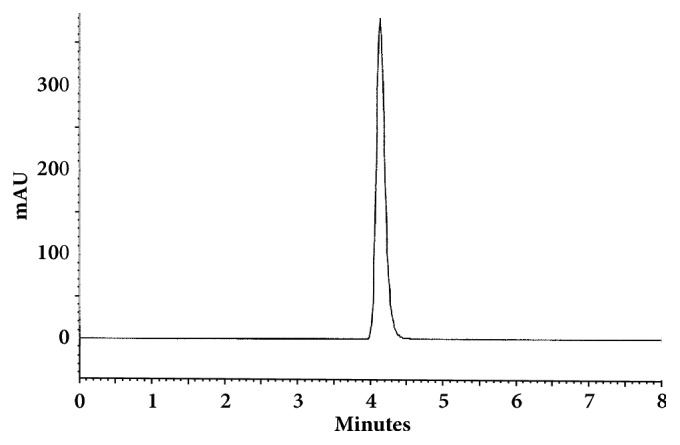
Chromatographic separation of loratadine. Chromatographic conditions described in Table 2.

**Figure 11:**
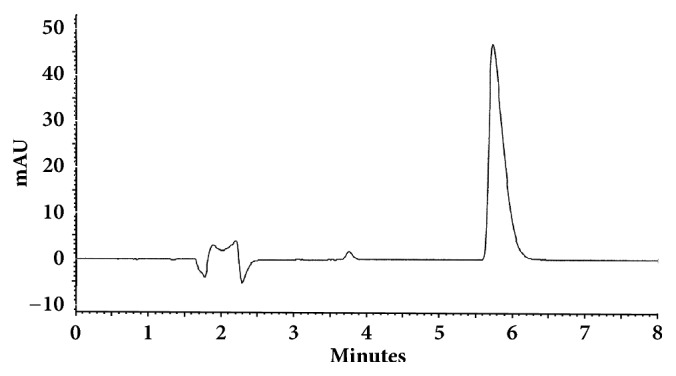
Chromatographic separation of olopatadine HCl. Chromatographic conditions described in Table 2.

Figures 5–11 show representative chromatograms of AZE, DESL, EB, FEX, KET, LOR, and OLO. All separations were obtained using a RP C-18 LUNA (150x4.6mm, 5 *μ*m) column. The mobile phase consisted of acetonitrile and acidified water (pH 2.1) in the following proportions: 15:85, v/v for desloratadine, 25:75, v/v for ketotifen and olopatadine, 32:68, v/v for fexofenadine, 35:65, v/v for azelastine and loratadine, and 45:55, v/v for ebastine. The flow rate was 1.0 ml min^−1^ and UV detections were made at 220 nm (FEX), 254 nm (EB), 271 nm (LOR), 279 nm (DESL), 287 nm (AZE), and 298 nm (KET and OLO).

### 3.3. Evaluation of Greenness of the Proposed Methods

As stated earlier, separation of seven antihistaminic drugs was far better with acetonitrile as compared to other organic solvents tested. Thus, we estimated the greenness of the proposed methods based on the GAC principles, described in the literature [18, 19, 32, 34]. A semiquantitative eco-scale was used to evaluate the greenness of proposed methods. Out of twelve GAC principles, five critical parameters dedicated to analytical methods were used and penalty points were assigned for each adverse factor.  Table 3 shows total eco-scale scores for proposed methods. Since the eco-scale value is larger than 75, the proposed methods can be considered excellent in terms of greenness [18, 19].

### 3.4. Method Validation for AZE

*Selectivity*. The excipients of the AZE nasal solution do not interfere in the main peak. Besides, the chromatographic peak purity index of AZE standard and sample solutions were evaluated based on photodiode array detector. The three-point peak purity index of AZE reference standard and sample solutions were identical (≥ 0.99). The results support the specificity of the proposed method.

*Linearity*. The proposed method was found to be linear in the concentration range from 10.0 to 60.0 *μ*g.mL^1^ of AZE. There was a linear correlation amongst AZE concentrations and corresponding peak area (R2 = 0.9960); besides the standard deviation of the residuals was 1.4%. Relevant data on linearly of proposed methods is presented in Table 4.

*Precision*. The method precision was accessed by repeatability (intra-day) and as intermediate precision (interday) tests. The AZE sample solutions (40.0 *μ*g mL^−1^) were prepared fresh and injected into the system (n=10), on three consecutive days.  Table 5 shows found amounts with respective variations (RSD). The RSD amongst results was below 2%. The obtained results suggest a good precision for the determination of AZE [23, 24, 35].

The recovery of the proposed method was accessed at three concentration levels (low, intermediate, and high).  Table 6 shows acceptable recovery of azelastine reference standard from the sample matrix. The results show a good accuracy of the proposed method [23].

*Detection and Quantitation Limits*. The estimated values of DL and QL were 1.10 and 3.32 *μ*g mL^−1^, respectively (Table 4). The QL was cross-checked by actual analysis of standard solution equivalent to 3.3 *μ*g mL^−1^ (n=3). The RSD was below 1.62%, far below recommended values [21, 24, 35].

The robustness of proposed method for analysis of AZE was evaluated by deliberate change in mobile phase flow rate (0.9, 1.0, and 1.1 mL mL^−1^), injection volume (18, 20, and 22uL), and mobile phase organic content (ACN 33, 35 e 37%). There was an insignificant impact on peak retention, peak area, and peak asymmetry (Figures 1–4).

## 4. Chromatographic Method for Separation of Azelastine Impurities

The chromatographic separation of azelastine and impurities B, D, and E was obtained using the Luna C18 column (150 × 4.6mm x 5 *μ*m). The mobile phase was composed of solution A (ACN with 0.1% formic acid) and solution B (purified water with 0.1% formic acid). The gradient system was 25 to 75% of solution A in 15 min. The mobile phase flow rate was kept constant at 1.0 mL min^−1^, and UV detections were made at 228 nm. The volume of injection was fixed at 20 *μ*L. All analyses were done at room temperature, and the column temperature was controlled at 25°C ± 1.

The optimum conditions for AZE and related impurities B, D, and E were fixed based on official system suitability parameters, peak asymmetry <2.0, and peak resolution > 2.0 [7, 12, 21]. The AZE nasal solution sample (250 *μ*g mL^−1^) was spiked with AZE impurities B, D, and E (10 *μ*g mL^−1^, each) and injected into the system.

**Figure 12:**
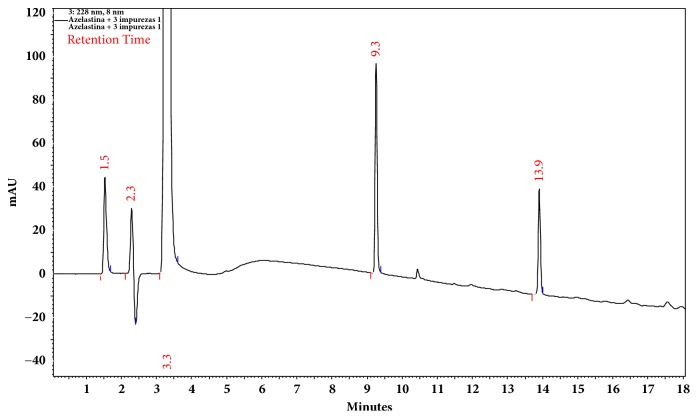
Chromatographic separation of azelastine HCl (3.3 min), impurity B (1.5 min), impurity D (9.3 min), and impurity E (13.9 min).

The system suitability parameters are described in Table 7, and representative chromatographic separation of AZE and related impurities B, D, and E is presented in Figure 12.

The selectivity of the method was accessed by peak purity (>0.99) and relative retention (k') of impurity B (-0.35), AZE B (0.43), impurity D (3.04), and impurity E (5.04) standard solutions, separately.

The system suitability parameters such as peak resolutions between adjacent peaks and peak symmetry were according to the specifications for separation of azelastine and its related impurities described in official compendiums [7, 12, 16].

## 5. Conclusions

The proposed ecologically correct methods may contribute in high-throughput analysis of seven antihistaminic drugs, with total organic residue < 2.5 mL/analysis. The proposed method for separation of azelastine and related organic impurities B, D, and E is highly efficient and economical. The total analysis time was 15 minutes and the mobile phase is free of ion pairing agents and buffer salts. All separations were obtained on a conventional reverse phase HPLC systems and can be considered excellent in terms of greenness. The proposed methods may subsidize in the evaluation of quality, efficacy, and safety of antihistaminic drugs.

## Figures and Tables

**Table 1:** The chemical names, molecular structures, and dissociation constants of azelastine HCl, desloratadine, ebastine, fexofenadine HCl, ketotifen, loratadine, and olopatadine HCl [17].

Antihistamines	Chemical name	pKa	Chemical structure
Azelastine HCl	4-[(4-chlorophenyl)methyl]-2-(1-methylazepan-4-yl)phthalazin-1-one hydrochloride	8.88	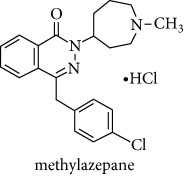

Desloratadine	8-Chloro-6,11-dihydro-11-(4-piperidinylidene)-5H- benzo[5,6]cyclohepta[1,2,b]pyridine	4.33 and 9.73	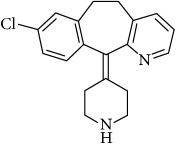

Ebastine	1-[4-(1,1-Dimethylethyl)phenyl]-4-[4-(diphenylmethoxy)-1-piperidinyl]-1-butanone	8.43	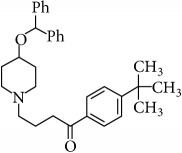

Fexofenidine HCl	2-[4-[1-hydroxy-4-[4-[hydroxy(diphenyl)methyl]-1-piperidyl]butyl]phenyl]-2-methyl-propionic acid hydrochloride	4.04 and 9.01	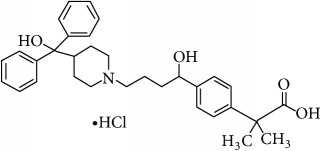

Ketotifen	4-(1-Methyl-4-piperidinylidene)-4,9-dihydro-10H-benzo[4,5]cyclohepta[1,2-b]thiophen-10-one	7.18 and 8.11	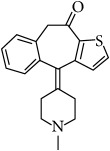

Loratadine	Ethyl 4-(8-chloro-5,6-dihydro-11H-benzo[5,6]cyclohepta[1,2-b]pyridin-11-ylidene)-1-piperidinecarboxylate	4.33	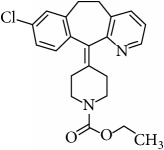

Olopatadine HCl	(11Z)-11-[3-(Dimethylamino)propylidene]-6,11-dihydrodibenz[b,e]oxepin-2-acetic acid hydrochloride	3.78 and 9.76	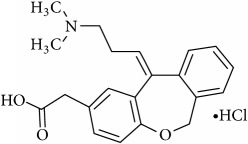

**Table 2:** Optimum chromatographic conditions for separation of seven antihistaminic drugs.

Drug	Mobile Phase ACN:H2O (pH 2.1)*∗*	Flow variation (0.8-1.2 mL min^−1^)*∗*	Injection volume variation (2 – 20 *μ*L)*∗*	Detection (nm)	Retention factor (k')	Peak Asymmetry (As)	Theoretical plates (N/m)
Azelastine HCl	35:65 (v/v)	1.0	20	287	1.52	1.34	14390

Desloratadine	15:85 (v/v)	1.0	20	275	1.26	1.32	15052

Ebastine	45:55 (v/v)	1.0	20	254	1.54	1.60	12698

Fexofenidine HCl	32:68 (v/v)	1.0	20	217	1.10	1.04	14720

Ketotifen	25:75 (v/v)	1.0	20	296	0.82	1.79	12512

Loratadine	35:65 (v/v)	1.0	20	271	1.26	1.38	15680

Olopatadine HCl	25:75 (v/v)	1.0	20	296	1.63	1.33	15412

*∗* Optimum value for each drug.

*∗∗*N/m: theoretical plates per meter.

**Table 3:** Eco-scale scores for proposed methods for analysis of FEX, EB, LOR, DESL, AZE, KET, and OLO (adopted from [18, 19]).

Factor	Penalty points
Reagents (HCl)	8

Acetonitrile	4

Instruments (HPLC)	1

Occupational hazards	3

Waste	8

Total penalty points	24

Analytical Eco-Scale total score	76

**Table 4:** Linear regression data in the analysis of AZE.

STATISTICAL PARAMETERS	
Concentration range (*μ*g mL^−1^)	10.0 – 60.0

Slope of the curve (*a*)	34754

Intercept (*b*)	91113

Correlation coefficient (R^2^)	0.9959

Standard deviation of the residuals	1.4%

DL (*μ*g mL^−1^)	1.10

QL (*μ*g mL^−1^)	3.32 (RSD < 2%)

DL= detection limit; QL= quantitation limit.

**Table 5:** The inter- and intraday precision data obtained in the analysis of AZE sample solutions (40.0 *μ*g.mL^−1^).

	Accessed AZE (*μ*g mL^−1^)
*Inter-day * **∗** **∗**	38.11 ± 0.58
RSD (%)	1.52
*Intra-day * **∗**	
Day 1	38.43 ± 0.54
RSD (%)	1.40
Day 2	38.26 ± 0.42
RSD (%)	1.10
Day 3	37.71 ± 0.54
RSD (%)	1.44

**Table 6:** Recovery of standard AZE added to fortify sample solutions and analyzed by the proposed method.

Sample	Added concentration (*μ*g mL^−1^)		Found concentration (*μ*g mL^−1^)	Recovery
	Sample	Standard	Standard	(%)
AZE	15.00	10.0	9.31 ± 0.12	93.10
	15.00	25.0	24.49 ± 0.22	97.97
	15.00	40.0	39.31 ± 0.18	98.27

**Table 7:** System suitability parameters obtained in the separation of azelastine and related impurities.

	Retention factor (k')	Peak resolution (Rs)	Peak asymmetry (As)
Impurity B	-0.35		1.36

Azelastine	0.43	9.0 (B-Aze)	1.36

Impurity D	3.04	17.14 (Aze-D)	1.14

Impurity E	5.04	13.14 (D-E)	1.05

k': relative retention (retention factor).

Rs: resolution between adjacent peaks.

## Data Availability

The data used to support the findings of this study are available from the corresponding author upon request.
